# Exploring the Cognitive Effects of Closed‐Serial and Closed‐Continuous Exercise in Aging: A Study on Dance and Endurance Training

**DOI:** 10.1002/ejsc.70196

**Published:** 2026-07-20

**Authors:** R. Forte, O. Caputo, C. Vecchiato, F. Sarto, G. Martino, G. Tedesco, M. Marino, A. Angrilli, G. De Vito, M. Narici

**Affiliations:** ^1^ Department of Movement Human and Health Sciences University of Rome “Foro Italico” Rome Italy; ^2^ Neuromuscular Physiology Laboratory Department of Biomedical Sciences University of Padova Padova Italy; ^3^ Department of General Psychology University of Padova Padova Italy; ^4^ Movement Control and Neuroplasticity Research Group KU Leuven Leuven Belgium

**Keywords:** executive functions, exercise complexity, exercise intensity, older adults, physical activity

## Abstract

Physical exercise benefits both cognition and physical health in aging, but it remains unclear whether benefits derive mainly from intensity or task complexity. Continuous exercises involve repetitive and predictable movements (e.g., running), whereas serial exercises (e.g., dancing) integrate varied motor sequences, and are cognitively more demanding. Clarifying the contribution of intensity and complexity is crucial for designing physically safe yet cognitively stimulating programs, maximizing preventive and rehabilitative potential in older adults. The aim of the study was to compare the ability of continuous versus serial exercises to improve executive functions. Seventy‐two participants (mean age 66.8) were assigned to three groups: dance (DNC), endurance (END), and inactive controls (INA). Executive functions were assessed through the Flanker, Corsi‐Block, and Trail Making tests. Groups were compared with MANOVA, followed by ANOVAs and LSD post‐hoc. A group effect emerged in the Flanker task (*p* = 0.006). END showed faster overall response times (RT) than DNC (*p* = 0.002) and INA (*p* < 0.001); DNC and INA did not differ. Incongruent RT tended to be faster in END versus DNC (*p* = 0.068) and was faster versus IAC (*p* = 0.003). For congruent RT, DNC and END outperformed IAC (*p* = 0.002; *p* = 0.001). Conflict cost was lower in DNC versus INA (*p* = 0.041) and END (*p* = 0.043), with no INA versus END difference. Continuous exercise enhanced general processing speed, whereas serial exercise appeared more effective for cognitive flexibility and selective attention. These findings support prior evidence to guide future large‐scale interventions aimed at optimizing exercise‐based cognitive training for older adults.

## Highlights

- Continuous exercise enhances processing speed in aging.
- Dance supports flexibility and selective attention.
- Exercise complexity shapes cognitive outcomes beyond intensity.
- Different exercise modes target distinct executive processes.

## 1 Introduction

The involvement of cognitive functions, particularly of the executive functions, during physical activity and exercise is well‐established. Theories of motor control, cognitive neuroscience, and exercise psychology indicate that the performance of motor tasks, especially goal‐directed actions requiring planning, sequencing, and inhibition rely on executive control (Diamond 2000; Koziol and Lutz 2013). Neurophysiological evidence shows that all purposeful behavior, including movement, involves executive coordination and that the prefrontal cortex acts as a temporal integrator, orchestrating motor actions based on internal goals, environment, and expected outcomes, reinforcing the claim that executive control is essential for motor function at all ages and particularly in older age (Yogev‐Seligmann et al. 2008; Diamond 2013; Fuster 2015; Russell and Arcuri 2015).

The exercise and cognition research further supports the involvement of cognitive, and particularly executive functions, in physical exercise across age groups. Acute exercise studies show that executive functions are stimulated during moderate‐to‐vigorous physical activity depending on exercise type, intensity and volume (Chang et al. 2012; Voss et al. 2020; Martini et al. 2024; Festa et al. 2023). Long‐term effects of physical exercise of different kinds, such as aerobic, strength, and coordination, on cognitive and executive functions have been largely described (Diamond 2015; Cai et al. 2024). While these insights apply across the lifespan, they are particularly relevant in the context of aging. As individuals grow older, executive functions such as working memory, inhibition, and cognitive flexibility tend to decline, contributing to decreased autonomy and quality of life (Yogev‐Seligmann et al. 2008; Diamond 2013; Forte et al. 2015). Given the growing number of older adults worldwide, identifying exercise strategies that can effectively support executive functions in the older population is of crucial importance. In this regard, the study of physical exercise with cognitive engagement has become a prominent tool for cognitive enhancement as demonstrated by the increasing literature in the field (Chang et al. 2012; Diamond 2015; Voelcker‐Rehage and Niemann 2013). However, important gaps remain in our understanding of how to optimally stimulate cognitive health both in the general population and in aging through exercise interventions (Voelcker‐Rehage and Niemann 2013; Diamond and Ling 2016; Zhang et al. 2023).

Prominent authors in the field of exercise and cognition suggest a paradigm shift from focusing mainly on the quantitative dimensions of exercise (frequency, intensity, duration) to considering its qualitative features such as complexity and variability (Pesce et al. 2019). Qualitative parameters such as movement speed, order, or direction, are more difficult to quantify and standardize and are less investigated despite the need for studies addressing characteristics beyond the quantitative domain of frequency intensity and duration (e.g., components of motor skills) (Diamond and Ling 2016).

As proposed in recent reviews (Zhu et al. 2020; Shi et al. 2022) a possible way forward in establishing qualitative exercise guidelines is to use the traditional framework and analyze cognitive exercise effects based on the environmental conditions of the activity (i.e., open or closed) and the organization of the movements involved (i.e., discrete, serial, continuous skills). Closed skill activities are performed in stable and predictable environment (i.e., jogging, cycling, weightlifting); open skills, conversely, are executed in changing environments that require continuous adaptation and learning in response to external cues such as interaction with opponents or objects under time and space constrains (i.e., basketball, martial arts). Discrete skills have a distinct beginning and end (e.g., kicking a ball); serial skills are discrete actions combined in a particular order to form a more complex sequence of movement (e.g., gymnastics routine); continuous skills involve the ongoing repetition of a movement pattern without evident beginning and end (e.g., running). Closed skills are generally less cognitively demanding than open skills, and continuous skills less complex than serial ones, as the latter require coordination of multiple movements and limbs. From a cognitive point of view, closed‐continuous skills are considered to require less mental manipulation processes than open‐serial. Such traditional taxonomy may help the “quantification” of the cognitive effort of the physical exercise and support the development of exercise prescription for cognitive benefits. A recent meta‐analysis (Zhu et al. 2020) on studies performed across age groups found that, cross‐sectionally, open skill exercise has an advantage over closed skill for inhibition and cognitive flexibility. However, the effects of the two types of exercises did not longitudinally differ, therefore only partially supporting the hypothesis that open skills are superior to closed skills in terms of cognitive benefits. Furthermore, the combination of continuous or serial actions, at least in children and adolescents, suggests that motor skills with open and/or closed attributes seem more helpful in improving executive functions (Shi et al. 2022). Similar evidence remains limited in older adults for whom exercise effects have mostly been studied by metabolic type (e.g., aerobic, strength, coordination) rather than organization of the actions involved.

Therefore, the present study was designed to provide further insights into how different closed‐skill exercise modalities influence executive functions in aging. Specifically, the study compared the impact on executive functions of recreational dance (as a closed‐serial activity) and endurance training (as a closed‐continuous activity); these activities were selected for their contrasting demands: endurance primarily targets aerobic efficiency which is known to indirectly support cognition, while dance primarily targets motor coordination and is known to directly supports cognition for its inherent features engaging memory, rhythm and multisensory integration. The final goal of the study was to fill the gap in the literature on exercise quality and provide a basis for future larger studies on the development of interventions and exercise guidelines for older adults. Based on the mentioned literature it was hypothesized that closed‐continuous exercise promotes greater cognitive efficiency, while closed‐serial exercise promotes cognitive flexibility.

## 2 Materials and Methods

The study followed the STROBE guidelines and has a quasi‐experimental cross‐sectional design. Participants were not randomly assigned to groups but were classified based on their habitual exercise practice, comparing older subjects practicing recreational dance, recreational endurance exercise and moderate physical activity.

The study received ethical approval by the committee of the local University (*n*. HEC‐DSB/15–2023) and was performed in accordance with the ethical standards as laid down in the 1964 Declaration of Helsinki and its later amendments.

### 2.1 Participants

Inclusion criteria were applied as follows: age over 60 years; medically stable health condition verified through a medical history questionnaire (Greig et al. 1994); absence of conditions that could affect variables of interest to the study (e.g., depression, uncontrolled cardiac illness, history of cerebrovascular disease, uncontrolled metabolic disease). Moreover, for the experimental groups: regular participation in either recreational dance or recreational endurance exercise (running, cycling, swimming) at least 4 h weekly for the previous 5 years; for the control group: practice of physical activity according to WHO “inactive” definition (i.e., < 150 min/week of moderate training or < 75 min/week of intense training) (WHO guidelines on physical activity, 2020) and no specific athletic history. After application of the above criteria, 72 male (*n* = 36) and female (*n* = 36) subjects aged between 60 and 78 (mean age 66.4 ± 4.1) were enrolled. Prior to participation to the study, all participants were described the aims, and the procedures involved in the study and signed an informed consent form. Three groups of participants were formed: the dance (DNC *n* = 24; mean age 67.42 ± 4.0), the endurance (END *n* = 24; mean age 65.2 ± 4.0) which were recruited from local dance and sport centers, respectively, and the inactive (INA *n* = 24; mean age 67.8 ± 4.1).

### 2.2 Testing

Following informed consent, participants underwent a battery of tests composed of the following tests. All tests were administered in a quiet room.

#### 2.2.1 Montreal Cognitive Assessment (MoCA)

The Italian version of the MoCA test was used to screen for mild cognitive impairment. The test is widely used and consists of 30 items assessing domains such as short‐term memory, language, orientation, visuospatial abilities, digit span, serial calculation, executive functions and attention. Administration is easy and rapid, taking about 10 min. A score of 26 or above is generally considered within the normal range (Nasreddine et al. 2005).

#### 2.2.2 Trail Making

The standard protocol of the paper and pencil trail making test was used to assess cognitive flexibility. Participants were required to draw lines connecting in ascending order and as quickly as possible 25 circles distributed over a sheet of paper (Sherman et al. 2023). The test has a part A with numbers only, and a part B with numbers and letters to be joined in alternation (i.e., 1‐A‐2‐B‐3‐C, and so on). The time taken to complete part A and part B (in seconds), and a summary score calculated by subtracting the time taken to complete part A from the time at part B (ΔTrail Making) were used for analysis.

The Psychology Experiment Building Language software (PEBL) was used to administer the subsequent tests (http://pebl.sourceforge.net/battery.html) (Mueller and Piper 2014).

#### 2.2.3 Corsi Block

The Corsi Block is a test of visuospatial working memory. In each trial nine blue squares were presented to the participants on a black computer screen. Squares flashed up (changed from blue to yellow) in a sequence, and participants were required, after the sequence was completed, to reproduce the exact order of flashing by selecting the squares by clicking with the mouse. The test started with a sequence of two flashing squares if participants responded correctly to two sequences of the same length, the sequence increased by one square for a maximum of 9. Only one incorrect attempt was allowed for each sequence, if two incorrect sequences were produced, the test was ended. Three practice trials that were not analyzed were given to participants. The length of the last correctly remembered sequence and number of correct trials were recorded to obtain Block span (maximum number of correctly remembered sequence of squares), Total Score (Block span x number of correct trials); Memory span (average length of correctly remembered sequence) was also calculated. Larger Total score reflected better performance. The test took approximately 5 min (Mueller and Piper 2014).

#### 2.2.4 Flanker Test

A modified version of the test The Eriksen Flanker (Mueller and Piper 2014) task was used to assess selective attention and inhibitory control. Participants were presented, on a black computer screen, a central target stimulus (e.g., an arrow) surrounded by flanking stimuli (e.g., other symbols) and instructed to respond to the direction of the central target by pressing the right or left shift key on the computer keyboard, ignoring distracting flanker surrounding it. The flanking stimuli could be congruent (matching the target e.g., <<<<<), incongruent (opposite to the target e.g., >><>>) or neutral (no other surrounding flankers or a different symbol e.g., 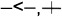), creating different levels of cognitive conflict. The task comprised 120 randomized trials, and the number of trials per condition was kept equivalent (40 trials each, 20 reps 2x3 design). A practice trial with the 8 possible conditions to familiarise the subject with the procedure preceded the test but was not used for analysis. The trial sequence started with a fixation cross displayed for 500 milliseconds (ms), then the target was presented in the middle of a black screen until a response was given or up to a maximum of 800 ms. The inter‐trial‐interval was 1000 ms. Measures of response time (RT, ms) and accuracy (ACC, percentage of correct responses) in both congruent and incongruent trials were taken and averaged for each condition and participant, with slower RT and reduced ACC in incongruent conditions reflecting the difficulty of inhibiting the response to the flanking stimuli. The difference in response times between congruent and incongruent conditions was used as an indicator of the strength of the interference effect, which is related to executive function.

### 2.3 Statistical Analysis

The data were initially screened for normality of distribution and outliers. After calculation of descriptive statistics, separate MANOVA was used to compare the participants allocated to the three groups (dance, inactive and endurance) on each set of cognitive measures (Flanker, Trail Making and Corsi block). In case of significant results, separate one‐way ANOVAs were planned as follow‐up, with subsequent post‐hoc comparison with LSD correction in case of significance. The level of significance was set at a value of *p* < 0.05. IBM SPSS version 29 was used for all computations.

## 3 Results

Data were found normally distributed. Following the calculation of the Mahalanobis distance four subjects were identified as outliers (two in the INA and two in the END group) and excluded from analysis. Therefore, the final sample of participants was 68. Tables 1, 2, 3 show the descriptive statistics of the study group.

**TABLE 1:** Mean and ±SD of age, anthropometric and cognitive characteristics.

	Dance (DNC *n* = 24)	Inactive (INA *n* = 22)	Endurance (END *n* = 22)
Age (years)	66.9 ± 4.1	67.4 ± 4.0	67.8 ± 4.1
Height (cm)	168,9 ± 7.5	169.3 ± 7.6	167.7 ± 9.1
Weight (kg)	71.6 ± 12.8	76.0 ± 14.0	63.0 ± 9.9^a^
BMI (kg/m^2^)	25.0 ± 3.9	26.4 ± 3.9	22.3 ± 1.8^b^
MoCA (score)	24.7 ± 2.7	24.0 ± 2.4	24.9 ± 3.0
Education (years)	11.0 ± 3.0	12.9 ± 3.0	11.7 ± 3.7

Abbreviation: MoCA = Montreal Cognitive Assessment.

^a^ *p* < 0.05 and significantly different from DNC and INA.

^b^ *p* < 0.001 and significantly different form DNC and INA.

**TABLE 2:** Means and ±SD of trail making and corsi block.

	Dance (*n* = 24)	Inactive (*n* = 22)	Endurance (*n* = 22)
Trail A (s)	39.9 ± 13.0	35.1 ± 13.8	39.9 ± 15.9
Trail B (s)	89.7 ± 33.9	79.9 ± 41.1	83.6 ± 45.2
ΔTrail (s)	49.8 ± 29.5	44.8 ± 33.0	43.7 ± 35.7
Block span (*n*)	4.6 ± 1.2	4.8 ± 1.0	4.8 ± 0.9
Total score	29.8 ± 14.9	34.6 ± 10.3	33.0 ± 12.0
Correct trials (*n*)	5.9 ± 1.1	7.0 ± 1.4	6.6 ± 1.7
Memory span (*n*)	3.9 ± 1.1	4.5 ± 1.0	4.3 ± 0.8

Abbreviations: Block span = maximum number of correctly remembered sequence of squares; Memory span = average length of correctly remembered sequence; Total Score = Block span x number of correct trials.

**TABLE 3:** Flanker's test results (means ± SD) with significant differences.

	Dance (DNC *n* = 24)	Inactive (INA *n* = 22)	Endurance (END *n* = 22)
Response time (ms)	544.9 ± 37.1^a^	551.3 ± 48.3	505.4 ± 40.3^b^
Accuracy (%)	0.9 ± 0.1	0.9 ± 0.1	0.9 ± 0.1
Total errors (n)	6.0 ± 5.9	7.5 ± 7.5	4.7 ± 2.4
Incongruent response time (ms)	582.8 ± 48.5	602.1 ± 52.7	556.3 ± 43.7^c^
Congruent response time (ms)	537.9 ± 41.0^**d**^	540.5 ± 49.2	494.7 ± 44.4^b^
Conflict cost (ms)	44.9 ± 30.1^**e**^	61.7 ± 20.6^**f**^	61.6 ± 29.4

*Note:* a = DNC vs. END^*^; b = END vs. INA^**^; c = END vs. INA*; d = DNC vs. END^**^; e = DNC vs. END^***^; f = DNC vs. INA^***^.

^*^ *p* < 0.01.

^**^ *p* < 0.001.

^***^ *p* < 0.05.

The MANOVA analyses performed on the sets of variables of the Trail Making and the Corsi Block tests revealed no statistically significant difference between groups, as evidenced by the values of Pillai's trace F (6,128) = 1.76, *p* = 0.11 and F (4,130) = 0,55, *p* = 0.70, respectively (Table 2). Regarding anthropometric variables, the MANOVA revealed a significant multivariate effect of group *F* (6, 128) = 2.63, *p* = 0.019, partial *η* ^2^ = 00.11. Univariate ANOVA showed that endurance participants significantly differed in weight (F (2,65) = 6.30, *p* = 0.003) and BMI (F (2,65) = 8.57, *p* < 0.001) from dance and inactive controls.

Regarding the set of variables of the Flanker's test, the MANOVA revealed a significant multivariate effect of group, *F* (8, 124) = 2.84, *p* = 0.006, partial *η* ^2^ = 00.15, indicating that 15% of the variance in the combined cognitive measures was explained by group differences. The observed power (0.94) suggests a high likelihood of detecting these effects in the present sample. Univariate ANOVAs revealed a significant effect of group on response times: total response time *F* (2, 65) = 7.78, *p* < 0.001, *η* ^2^ = 0.19; congruent response time, *F* (2, 65) = 7.30, *p* = 0.001, *η* ^2^ = 0.18; and incongruent response time, *F* (2, 65) = 4.97, *p* = 0.010, *η* ^2^ = 0.13. There was a trend towards significance for the conflict cost, *F* (2, 65) = 2.92, *p* = 0.061, *η* ^2^ = 0.08. No significant effect was observed for Accuracy and Total errors (Table 3).

**FIGURE 1:**
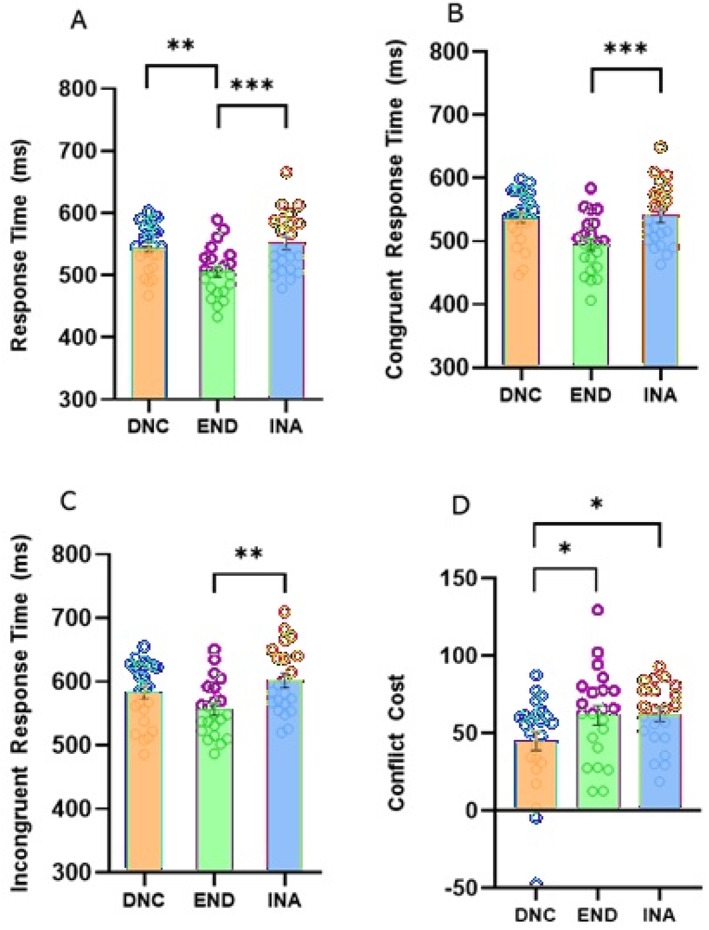
Response times (RT) and conflict cost (CC) in the different groups. Results are reported in panel A, B, C and D for Global RT, congruent RT, incongruent RT and CC, respectively (DNC = dancers, END = endurance, INA = inactive; ms = milliseconds; * = *p* < 0.05 ** = *p* < 0.01 *** = *p* < 0.001).

The post hoc comparisons revealed non‐significant differences between groups for the Total of Errors and accuracy (*p* > 0.05). For RT, the END group was significantly faster than the DNC (*p* = 0.002) and the INA group (*p* < 0.001), while non‐significant difference were observed between DNC and INA (*p* = 0.603). Similar results were found for incongruent RT, where the difference between END group and DNC showed a tendency to significance (*p* = 0.068), the DNC and the INA did not differ (*p* = 0.181) and the END subjects were significantly faster than the INA (*p* = 0.003). For congruent RT, no differences were observed between DNC and END (*p* = 0.844), while both the DNC and the END group were significantly faster than the INA (*p* = 0.002 and *p* = 0.001, respectively). Lastly for the conflict cost, the DNC subjects showed significantly lower values than the INA (*p* = 0.041) and the END (*p* = 0.043), with no significant differences between INA and END participants (*p* = 0.988) (Figure 1).

## 4 Discussion

The main findings of this study are that continuous exercise was associated with faster processing speed, whereas serial exercise showed a relative advantage in cognitive flexibility and selective attention. In general, the literature suggests that cognitive gains are more likely when training emphasizes complexity, novelty, and diversity (Moreau et al. 2015). Prior research indicates that not all physical activities afford equivalent cognitive benefits. In children, activities demanding coordination, rule‐following, and rapid decision‐making stimulate executive functions more than repetitive aerobic exercise (Diamond and Ling 2016; Best 2010). In adults, exercise programs integrating perceptual, motor, and cognitive challenges confer advantages over purely repetitive training (Moreau et al. 2015). In older adults, engagement in cognitively complex daily activities has been associated with long‐term preservation of executive functioning (Angevaren et al. 2007; Küster et al. 2016; Kobayashi et al. 2016).

A growing body of literature suggests that the cognitive impact of exercise in older adults depends not only on its metabolic profile (aerobic, resistance, coordination) but also on its qualitative structure (Voelcker‐Rehage and Niemann 2013; Moreau et al. 2015; Pesce 2012; Tomporowski and Pesce 2019). Aerobic endurance training has consistently been associated with improvements in global processing speed and attention, likely mediated by enhanced vascularization, angiogenesis, and neurotrophic support (Colcombe and Kramer 2003; Stillman et al. 2020). Conversely, dance and other complex activities appear to promote executive functions through different pathways, probably by promoting neural plasticity within frontal and parietal association networks supporting executive control and sensorimotor integration (Voelcker‐Rehage and Niemann 2013; Coubard et al. 2011; Kattenstroth et al. 2013). Their intrinsic demands, which include memorizing and reproducing motor sequences, synchronizing movements with rhythm and partners, integrating visual, auditory and proprioceptive cues engage frontal and parietal regions involved in working memory, cognitive flexibility, sensorimotor integration (Voelcker‐Rehage and Niemann 2013; Voelcker‐Rehage and Niemann 2013; Coubard et al. 2011) and cerebellum in timing and coordination (Stoodley et al. 2012). Indeed, dance interventions have been shown to improve flexibility, selective attention, and hippocampal plasticity in older adults (Rehfeld et al. 2017; Coubard et al. 2011; Kattenstroth et al. 2013).

Taken together, these findings emphasize the need to look beyond traditional metabolic classifications of exercise to consider structural dimensions such as continuity versus seriality of actions in the attempt to overcome the problem of quantification and of dose‐response determination (Pesce 2012; Tomporowski and Pesce 2019). Such a framework may capture subtle but meaningful differences in how physical activity stimulates the aging brain (Pesce 2012; Tomporowski and Pesce 2019; Klotzbier and Schott 2025), informing the design of interventions that are both effective and cognitively challenging. The present results support this theoretical framework, showing a significant multivariate effect of groups, with training type explaining 15% of the variance in executive measures. At the same time, the most meaningful cognitive variable, conflict cost, did not reach statistical significance. A post hoc power analysis (GPower) indicated adequate statistical power for the main response‐time outcomes (1–β ≈ 0.94), whereas power was lower for conflict cost (1–β ≈ 0.55), suggesting that this latter finding should be interpreted with caution and warrants further investigation in future studies.

Our findings indicate a general superiority of closed‐continuous endurance training in enhancing global processing speed and attentional efficiency. The END group showed better response times across both congruent and incongruent conditions reflecting a general enhancement of neural efficiencyin line with evidence that aerobic exercise promotes neurovascular adaptations, increases BDNF levels, and enhances synaptic efficiency, thereby improving information transmission and attentional control (Voelcker‐Rehage and Niemann 2013; Colcombe and Kramer 2003; Stillman et al. 2020). It should be noted that endurance participants had a significantly lower BMI than the other participants. Given the established associations between adiposity and cognition (Farruggia and Small 2019), additional analyses controlling for BMI were performed. The pattern of results remained unchanged confirming that this variable did not account for the observed cognitive differences.

Both endurance and dance showed improved performance under lower cognitive demands (congruent trials) with respect to inactive subjects, suggesting shared benefits for automatic processing. However, dance training showed a relative advantage in reducing conflict cost compared to endurance, a result that, although not statistically significant, aligns with previous studies highlighting the role of dance in enhancing cognitive flexibility and selective attention (Coubard et al. 2011). Dance presents unique characteristics that may help explain this observation. While classified as a closed skill of moderate intensity due to its structured environment and intermittent actions, dance is cognitively demanding as continuously requiring switching between physical and mental challenges. These demands likely stimulate and train executive processes, especially cognitive flexibility. Neuroimaging and behavioral evidence suggest that these benefits may be induced by motor and neural adaptations, such as enhanced visuo‐motor coordination, improved sensorimotor integration, and reduced inhibitory costs through greater prefrontal efficiency (Voelcker‐Rehage et al. 2011). In animal models, coordination training promotes synaptogenesis and angiogenesis, reinforcing the plausibility of such effects [ (Voelcker‐Rehage and Niemann 2013), (Voss et al. 2013)].

## 5 Limitations and Future Directions

A crucial limitation concerns exercise intensity. Endurance training typically entails continuous, higher‐intensity activity, while dance involves intermittent bouts of variable intensity. It is therefore difficult to disentangle the contribution of exercise type from that of exercise intensity. Notably, many endurance participants were cyclists, a subgroup known to show particularly high attentional performance (Pesce et al. 2011), which may have influenced results. Similarly, and likely due to the practice of exercise of high intensity, endurance participants had lower BMI than the other groups, a factor known to influence cognition through vascular and inflammatory mechanisms (Farruggia and Small 2019). However, controlling for BMI did not alter the cognitive results, suggesting that group differences are not explained by body composition. Future studies should nonetheless account for adiposity‐related variables when designing exercise–cognition interventions. Furthermore, the control group was not fully sedentary but moderately active, which could have attenuated between‐group differences, given that even habitual daily activity supports cognition in aging (Angevaren et al. 2007; Küster et al. 2016; Kobayashi et al. 2016). Importantly, education level did not differ substantially among groups, making it unlikely that this factor contributed to the observed cognitive effects. Another point concerns the lack of group differences in accuracy measures. This may reflect a ceiling effect due to the relative simplicity of the tasks, with speed rather than correctness being more sensitive to exercise‐related effects. Future studies should include tasks with higher cognitive load or multiple executive outcomes to better capture exercise‐induced changes.

## 6 Practical Implications

From an applied perspective, the findings highlight the potential for tailoring exercise programs to specific cognitive outcomes. Endurance training, through its vascular and neurotrophic benefits, may be particularly suitable for maintaining general processing speed and attentional vigilance, capacities that support everyday activities requiring sustained alertness. Dance, or other serial activities, by contrast, may be strategically used to train cognitive flexibility and adaptability, which are essential skills for coping with novel or dynamic environments. Importantly, dance also incorporates social and emotional dimensions, which may enhance adherence and provide additional cognitive stimulation. These modalities may therefore be complementary, and future interventions could combine them within multimodal programs designed to address the heterogeneous needs of older adults in both preventive and rehabilitative contexts. The present work, though not introducing new concepts (i.e., open‐closed skills, serial, discrete continuous skills), suggests a practical method to quantify the intensity of coordination exercise for use in prescription guidelines, and thus a model for incorporating qualitative features of exercise complementing those of intensity and volume.

## 7 Conclusions

The present findings support the hypothesis that different types of closed‐skill exercise exert differential effects on executive functions in older adults. Endurance training appears to enhance global processing speed, while dance shows promise for improving cognitive flexibility and selective attention. These results must be interpreted considering the sample size and study design, and the presence of potential confounding factors such as exercise intensity and baseline activity levels. Nevertheless, they point to meaningful directions for future large‐scale investigations and provide a rationale for designing exercise‐based cognitive interventions that move beyond the traditional aerobic–strength–coordination classification, emphasizing the role of motor complexity and task structure.

## Author Contributions

R. F., A. A., G. D. V., M. N.: conceptualization, methodology. R. F., O. C., C. V., F. S., G. M., G. T., M. M.: investigation, data curation, formal analysis. R. F.: writing‐ original draft preparation. R. F., M. M., A. A., G. D. V., M. N.: reviewing and editing. M. N.: resources, funding acquisition, project administration. A. A., G. D. V., M. N.: supervision.

## Funding

Next Generation EU (DM 1557 11.10.2022)–National Recovery and Resilience Plan, Investment PE8–Project Age‐It: ‘Aging Well in an Aging Society’. The views and opinions expressed are only those of the authors and do not necessarily reflect those of the European Union or the European Commission. Neither the European Union nor the European Commission can be held responsible for them.

## Ethics Statement

The study received approval by the board for research of the local university.

## Consent

All participants signed informed consents before the assessments.

## Conflicts of Interest

The authors declare no conflicts of interest.

## Human and Animal Rights

The study has been performed in accordance with the ethical standards as laid down in the 1964 Declaration of Helsinki and its later amendments.

## Data Availability

The data that support the findings of this study are available on request from the corresponding author. The data are not publicly available due to privacy or ethical restrictions.
